# Spontaneous Perforation of Common Bile Duct: A Rare Presentation of Gall Stones Disease

**DOI:** 10.1155/2016/5321304

**Published:** 2016-06-28

**Authors:** Duminda Subasinghe, Edippuli Arachchige Don Udayakumara, Upul Somathilaka, Milinda Huruggamuwa

**Affiliations:** Department of General Surgery, General Hospital, 60000 Kurunegala, Sri Lanka

## Abstract

*Background*. Spontaneous perforation of the extrahepatic biliary system is a rare presentation of gall stones. Very few cases of bile duct perforation have been reported in adults. It is rarely suspected or correctly diagnosed preoperatively.* Case Presentation*. A 66-year-old female presented at the surgical emergency with 3 days' history of severe upper abdominal pain with distension and repeated episodes of vomiting, as she had evidence of generalized peritonitis and underwent an exploratory laparotomy. A single 0.5 cm × 0.5 cm free perforation was present on the anterolateral surface of the common bile duct at the junction of cystic duct. A cholecystectomy and the CBD exploration were performed.* Conclusion*. Spontaneous perforation of the extrahepatic bile duct is a rare but important presentation of gall stones in adults. Therefore, awareness of the clinical presentation, expert ultrasound examination, and surgery are important aspects in the management.

## 1. Introduction

Spontaneous perforation of the wall of the extrahepatic or intrahepatic bile duct with biliary peritonitis is a rare event in infants and an extremely rare event in adults. It was first described in 1882 by Freeland [1]. Generalized biliary peritonitis following perforation of the biliary tract is commonly a sequel of rupture of an inflamed gangrenous gall bladder [2]. Bile duct perforation is relatively common in infants [3, 4] and is related to congenital biliary system anomalies. The aetiology of biliary tract perforation in the adults is commonly attributable to intramural infection, necrosis of the wall of the bile duct secondary to thrombosis, increased intraductal pressure secondary to obstruction, cirrhosis, and direct erosion by calculi.

The presentation of biliary peritonitis varies and because of its rarity the correct preoperative diagnosis is often difficult and delayed. This along with the associated comorbidity of mostly the elderly, patients in whom it is seen, results in a mortality rate of 30–50% [4]. We report an interesting, rare case of spontaneous extrahepatic bile duct perforations with choledocholithiasis, in an adult female. Its clinical presentations, investigative findings, and management are discussed and relevant literatures are reviewed. The rarities of this case are the atypical site of CBD perforation and its occurrence at a relatively older age.

## 2. Case Presentation

A 66-year-old female presented at the surgical emergency with 3 days' history of severe upper abdominal pain with distension and repeated episodes of vomiting. The abdominal pain increased in intensity and became continuous and more diffuse for the last two days. Her past medical, surgical, and family histories were unremarkable. General examination revealed that she was dehydrated, febrile (101 F), and having a pulse rate of 120/minute and blood pressure of 90/70 mm of Hg. She was not icteric and free of cervical lymphadenopathy. Her abdomen was distended with guarding and rebound tenderness mainly in the upper abdomen. There was no evidence of free fluid in the abdomen. Liver dullness was not obliterated. Bowel sounds were absent.

Hematological investigations revealed neutrophil leukocytosis [17700 cells/mm^3^ (84% neutrophil)] with normal total bilirubin [10.2 *μ*mol/L (5–21)], alkaline phosphatase [255 IU/L (70–360)], and alanine transaminase [14 IU/L (8–38 IU/L)]. Serum amylase level was normal. Her erect chest radiograph was normal without evidence of pneumoperitoneum but on USS abdomen showed small amount of free fluid in hepatorenal pouch. Therefore, definite preoperative diagnosis was not possible. As she had evidence of generalized peritonitis, she underwent an exploratory laparotomy, after adequate resuscitation and under broad spectrum antibiotic coverage.

**Figure 1:**
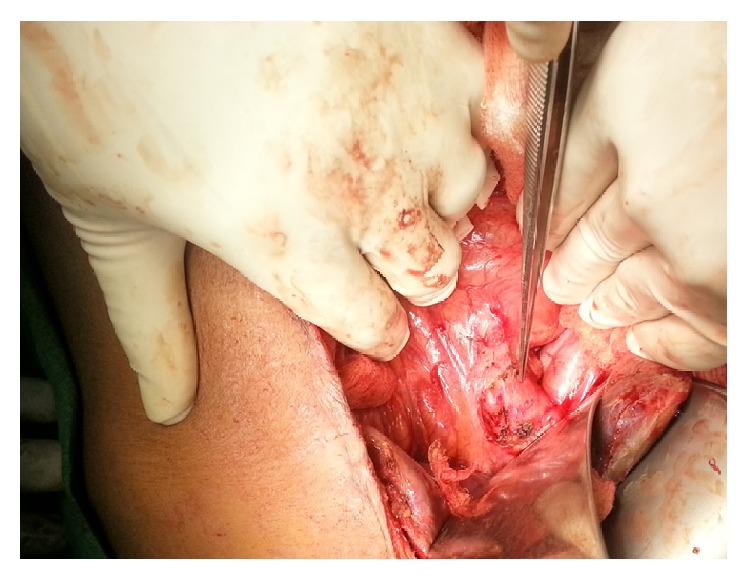
Perforation on the anterolateral surface of the common bile duct.

Operative findings included bilious peritoneal fluid in dependent parts of the peritoneal cavity with localized collection of bile inside the lesser sac. Gall bladder was distended with thickened wall and contained multiple stones. A single 0.5 cm × 0.5 cm free perforation was present on the anterolateral surface of the common bile duct at the junction of cystic duct (Figure 1). The common bill duct was dilated and contained multiple stones. A cholecystectomy was done and the CBD was explored. Multiple small stones were removed, irrigation was done with warm normal saline, and the CBD was closed over a T-tube inserted through the site of perforation. The peritoneal cavity was thoroughly irrigated with warm normal saline. The abdomen was closed after inserting a suction drain in the hepatorenal space. Unhealthy edges of the perforation site were excised and sent for the histology which subsequently showed inflamed necrotic tissue without any evidence of malignant cells. The histology of the gallbladder was suggestive of chronic cholecystitis.

**Figure 2:**
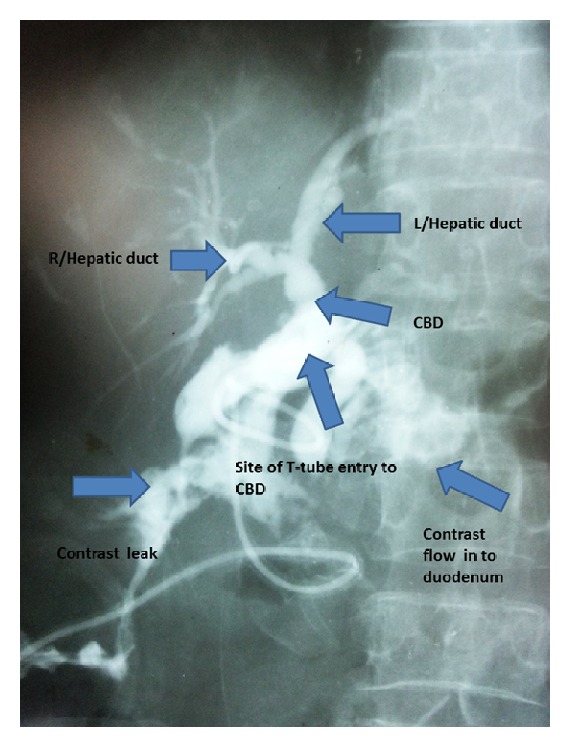
Postoperative T-tube cholangiogram showing biliary anatomy and contrast leak.

The patient made a slow postoperative recovery. There was persistent drainage of bile through the subhepatic drain until the 10th postoperative day. T-tube cholangiogram performed on the 10th postoperative day showed leaking and the pooling of contrast at T-tube insertion site of CBD with leaking tract extending towards drain site (Figure 2). It also showed that there was a filling defect in the distal CBD with contrast flow into the duodenum. Therefore, subsequently she underwent endoscopic retrograde cholangiopancreatography and extraction of distal CBD stone. T-tube was removed on the 30th postoperative day and she was discharged on 35th postoperative day. She was doing well at the time of the first visit and on follow-up visits until 1 year.

## 3. Discussion

Spontaneous perforation of common bile duct is a rare cause of biliary peritonitis. It is rarely suspected or diagnosed preoperatively. Perforation of the biliary system is a recognized complication of cholelithiasis; the diagnosis should be suspected if a perihepatic abscess or peritonitis is combined with biliary stone disease. Kang et al. [5] reviewed 70 cases of spontaneous bile duct perforations in adults. Among these, 42 patients had perforation in common bile duct, followed by hepatic duct in 28 cases. Although the pathogenesis of spontaneous biliary perforation is poorly understood, the commonest cause for perforation was a stone in adults. The recognized mechanisms include calculus perforation at the site of impaction and erosion. The other causes for spontaneous perforation of bile duct reported in literature were increased canalicular pressure due to obstruction by tumour, spasm of the sphincter of Oddi, intramural infection, mural vessel infarction leading to mural necrosis, or rupture of a biliary tract anomaly such as cyst or biliary diverticulum, a connective tissue defect, or ischemic compromise that results in perforation of the duct wall and previous biliary tract surgeries [6–9]. Sometimes it can be idiopathic. Talwar et al. [10] have reported a case of CBD perforation during pregnancy and the site of the perforation was supraduodenal portion of the CBD with stone impaction. Our patient's ultrasound abdomen showed perihepatic fluid collection suggesting a diagnosis of perforated viscus but there gall stones were not visualized. It is important to mention that ultrasound scan of the abdomen is an operator dependent imaging modality. Therefore, our patient's preoperative diagnosis was uncertain and therefore the definitive diagnosis was made intraoperatively.

Perforation of the common bile duct was most probably related to the abrupt increase in local intraluminal pressure causing erosion of the stones. This results in abrupt increase in intraluminal pressure and decreased blood flow in the vessels which run along the lateral border of the bile ducts, resulting in ischemia on the anterior surface of the bile duct [6]. This might explain the site of CBD perforation in our patient. The commonest reported site of perforation is at the junction of the cystic and hepatic ducts [8, 11] and our patient's findings were compatible with previous literature. This patient presented with a 3-day delay with generalized peritonitis. Facilities for the intraoperative cholangiogram or ERCP were not available. Recommended treatment in this situation is T-tube drainage of the common bile duct along with cholecystectomy. In cases with distal obstruction of the CBD, a biliary enteric bypass should be done. Primary suture repair of the CBD is considered if a preoperative cholangiogram is available and it shows no pathology distal to the perforation. Therefore, in the absence of intraoperative cholangiogram, this patient's condition was best managed by closure over a T-tube [12] followed by postoperative ERCP in a specialized hepatobiliary center [12]. The mainstay of the management of patients with suspected stones in the common bile duct has three aims: to evaluate the probability of stones in the common bile duct, to treat these stones when present, and to treat the source of the stones, gallbladder. The most common treatment modality is ERCP, with duct cannulation and clearance rates reaching 98% in expert hands [13]. Laparoscopic common bile duct exploration and clearance of stones were found to be as effective as preoperative and postoperative ERCP, with no significant difference in morbidity and mortality [14].

## 4. Conclusion

In conclusion, spontaneous perforation of the extrahepatic bile duct is a rare but important presentation of gall stones but important condition in adults. Therefore, awareness of the clinical presentation and expert ultrasound examination are important adjuncts in the diagnosis. Conservative surgery is the mainstay of treatment in the acute presentation.

## Abbreviations

CBD: Common bile duct
USS: Ultrasound scan
ERCP: Endoscopic retrograde cholangiopancreatography.
